# Preliminary results of the EPIDIA4Kids study on brain function in children: multidimensional ADHD-related symptomatology screening using multimodality biometry

**DOI:** 10.3389/fpsyt.2025.1466107

**Published:** 2025-03-17

**Authors:** Yanice Guigou, Alexandre Hennequin, Théo Marchand, Mouna Chebli, Lucie Isoline Pisella, Pascal Staccini, Vanessa Douet Vannucci

**Affiliations:** ^1^ R&D Lab, O-Kidia, Nice, France; ^2^ Bioelectronic Lab, Ecole des Mines de Saint-Étienne, Gardanne, France; ^3^ Unité propre de recherche (UPR) Risk Epidemiology Territory INformatics Education and Health (UPR RETINES), Université Côte d’Azur, Nice, France; ^4^ Medical Information Department, Alpes-Maritimes Hospitals Group (GHT 06), Nice, France

**Keywords:** digital epidemiology, multidimensional assessment, biometry, multimodality, child development, ADHD, Cognitive and behavioral performances

## Abstract

Attention-deficit hyperactivity disorder (ADHD) occurs in 5.9% of youth, impacting their health and social conditions often across their lifespan. Currently, early diagnosis is constrained by clinical complexity and limited resources of professionals to conduct evaluations. Scalable methods for ADHD screening are thus needed. Recently, digital epidemiology and biometry, such as the visual, emotional, or digit pathway, have examined brain dysfunction in ADHD individuals. However, whether biometry can support screening for ADHD symptoms using a multimodal tech system is still unknown. The EPIDIA4Kids study aims to create objective measures, i.e., biometrics, that will provide a comprehensive transdiagnostic picture of individuals with ADHD, aligning with current evidence for comorbid presentations. Twenty-four children aged 7 to 12 years performed gamified tasks on an unmodified tablet using the XAI4Kids^®^ multimodal system, which allows extraction of biometrics (eye-, digit-, and emotion-tracking) from video and touch events using machine learning. Neuropsychological assessments and questionnaires were administered to provide ADHD-related measures. Each ADHD-related measure was evaluated with each biometric using linear mixed-effects models. In contrast to neuro-assessments, only two digit-tracking features had age and sex effects (p < 0.001) among the biometrics. Biometric constructs were predictors of working memory (p < 0.0001) and processing speed (p < 0.0001) and, to a lower extent, visuo-spatial skills (p = 0.003), inattention (p = 0.04), or achievement (p = 0.04), where multimodalities are crucial to capture several symptomatology dimensions. These results illustrate the potential of multimodality biometry gathered from a tablet as a viable and scalable transdiagnostic approach for screening ADHD symptomatology and improving accessibility to specialized professionals. Larger populations including clinically diagnosed ADHD will be needed for further validation.

## Introduction

Attention-deficit hyperactivity disorder (ADHD) is one of the most common neurodevelopmental disorders, affecting approximately 5% [95% confidence interval (CI) = 5.0, 5.6] and 7.2% (95% CI = 6.7, 7.8) of children and adolescents across the world (1, 2). This disorder is characterized by developmental impairments of inattention, hyperactivity, or a combination of both. Various genetic and environmental risk factors contribute to ADHD (e.g., prenatal factors and early life adversities) (3), but the pathomechanism of such a condition is not yet deciphered (4).

Youth with ADHD are affected in their daily cognitive, behavioral, and social functions (5) and often require support in and beyond school in many trans-nosographic dimensions such as emotion dysregulation, irritability, and impairment in executive functions (6). Longitudinal studies have shown that persistent symptoms in childhood and adolescence lead to chronic conditions that may persist into adulthood (7). Youth with ADHD have a higher risk for somatic, medical, and psychiatric conditions (4, 6) with a decreased life expectancy of 10 to 15 years compared to the general population (1, 8). Therefore, ADHD has become a public health challenge with important socio-economic burdens to children, families, and the whole society.

The early detection and diagnosis of ADHD is reliable (4) when evaluated by well-trained practitioners following the standard diagnostic criteria (9). Adequate early detection of ADHD and its associated conditions appears crucial to reduce the risk of comorbidities, alter the course of the disorder, and hereby improve several health and well-being outcomes of children and adolescents (10–12). However, access to ADHD evaluation and diagnosis is limited by a lack of consultation time, trained specialists in the current care system (1, 13, 14), and stigmatization for both patients and families (15, 16). A further complementary approach based on technology (17) has emerged to reach screening at a large scale while improving diagnosis accuracy and lowering stigma and cost (18). Objective screening methods are thus increasingly needed; however, they have to be translated into clinical practice after rigorous scientific validation.

Digital epidemiology relies on the data acquired from devices to advance the understanding of health and disorders related the population dynamics (19). In the meantime, lab-scale evidence showed that biometry such as eye-tracking (20–23), digit-tracking (24, 25), or emotion dysregulation (26–28) has at the potential to provide high accuracy classification of ADHD through digital assessments (29). Thus, biometry can measure at once multiple traits (iris, fingerprints, face, retina, hand geometry, and voice) of an individual, which will allow the identification of various dimensions and comorbid presentations as symptoms of ADHD. In contrast to neuroimaging modalities (30, 31) or physiological signals (i.e., electroencephalogram and electrocardiograms) (32, 33), biometry markers or biometrics can easily be used in real-life practice or even remotely during ADHD assessments in children and adolescents. However, very few diagnostic biomarkers for ADHD have been validated clinically (34, 35) based on criteria set by the World Federation of ADHD and the World Federation of Societies of Biological Psychiatry (4). Nevertheless, these results have no attempts to screen for ADHD symptomatology and its comorbidities in real-life practice. An alternative transdiagnostic approach using digital epidemiology may support a better understanding of brain function by providing a screening tool to the general population.

In this paper, we present the preliminary results of the EPIDIA4Kids study (36) based on a multimodality biometry system combining digit-, eye-, and emotion-tracking measurements to examine brain function in children aged 7 to 12 years. Using machine learning, we investigated whether multimodal biometry could serve as an objective measurement for ADHD-related symptomatology in a pediatric population.

## Methods

### Ethics approval

EPIDIA4Kids is an observational uncontrolled multi-center study (several cities in France) approved by the Committee for the Protection of Individuals Sud-Est II under the national French register number 2022-A00766-37 and the Commission Nationale de l’Informatique et des Libertés (CNIL). This study was registered in October 2022 on ClinicalTrials.gov under the number NCT05577533. The study is designed to create a brain function normative database relying on digital epidemiology in children aged 7 to 12 years using multimodality biometry (36).

### Participants

The present study is a preliminary investigation aimed at exploring the feasibility and potential of using multimodal biometry for ADHD symptomatology screening. As such, it is part of a larger ongoing study that aims at recruiting 400 children for a power analysis of 0.8, a confidence level of 0.05, and a type I error, based on three dimensions of executive skills, processing abilities, and processing speed, previously reported in the literature (36). Twenty-four children aged 7 to 12 years were enrolled in the EPIDIA4kids study from March 2023 to December 2023. Details on recruitment and participants’ characteristics were reported previously. Informed consent was obtained from parent/legal representatives with child assent. Children were screened to ensure that they have no parent-reported history of major psychiatric or neurological disorders, brain injury, or other medical conditions that would affect their brain development. Children born prematurely (<32 weeks gestational age) with significant prenatal drug or alcohol exposure were also excluded.

### Questionnaires and neuro-assessments

All questionnaires and neuro-assessments are detailed in **Table 1**. Children performed the digitalized Wechsler Intelligence Scale for Children^®^ Fifth Edition (WISC-V) battery (1.5 hours; 37). Parents or legal representatives answered standardized questionnaires digitally.

**Table 1:** The description of each target variable and each candidate for explanatory variables.

Target variable		Neuropsychopathological measure
***Neuro-assessments (WISC-V)***	Block design	Visuo-spatial subtest
	Matrix reasoning	Fluid reasoning
	Digit span	Working memory
	Letter–number sequencing	Auditory verbal working memory
	Cancellation	Visuo-spatial processing speed
	Coding	Processing speed
	Symbol search	Processing speed, impulsivity
	Comprehension	Comprehension
	Similarities	Verbal comprehension
	Vocabulary	Verbal comprehension
***Questionnaires***	Hyperactivity/impulsivity	ADHD scale, an 18-item self-report questionnaire designed to assess attention-deficit hyperactivity disorder (ADHD)
	Inattention	
	Social problems	Social Problems, Child Behavior Checklist. The Child Behavior Checklist (CBCL) is a widely used questionnaire to assess behavioral and emotional problems
	Achievement	Grit Scale for Children and Adults (GSCA) assesses the ability to sustain a focused effort to achieve success in a task, regardless of the challenges that presented themselves, and the ability to overcome setbacks in children and adults
	Manual laterality	The Edinburgh Handedness Inventory (EHI) is a measurement scale used to assess the dominance of a person’s right or left hand in everyday activities.
Explanatory variables (biometric features)		Description/parameter or model
***Digit-tracking***	Touch speed	Mean of point-by-point trajectory speed (in pixels per second)
	Touch area	Area occupied by a gesture, computed as the area occupied by a minimal adaptive polygon fitted to the gesture (in pixels squared)
	Touch distance	Sum of point-by-point trajectory distance (in pixels)
	Touch duration	Duration of a touch gesture (in seconds)
	Touch height	Maximum value of height (Y-axis absolute) gesture distance (in pixels)
***Eye-tracking***	Angular distance	Distance between two consecutive gaze angles (in degrees) $=\cos^{-1}\left(\frac{a.\;b}{\left|a\right|.\left|b\right|}\right)$
	Total angular distance	Sum of absolute distances between consecutive gaze angles (in degrees) $\sum^{}\left|\alpha \right|$
	Angular velocity	Angular distance divided by the time elapsed between two consecutive gaze angles (in degrees per second) $\frac{d\alpha }{dt}$
	Saccade/fixation	Boolean indicator whether the eye is performing a saccade or a fixation in each frame according to a fixed velocity threshold where fixations are segments with point-to-point velocities below the set velocity threshold, and saccades are segments with velocities above this threshold [the velocity-threshold identification (I-VT)]. In the present study, the velocity threshold of 100°/sec was applied and a fixation duration of 70 ms
***Emotion***	7 emotions: anger, disgust, fear, happiness, neutral, sadness, and surprise	Probability distribution that the emotion is present in the video frame (between 0 and 1)

CBCL, Child Behavior Checklist; ADHD, attention-deficit hyperactivity disorder; GSCA, Grit Scale for Children and Adults; WISC-V, Wechsler Intelligence Scale for Children^®^ Fifth Edition; EHI, Edinburgh Handedness Inventory.

### XAI4Kids^®^ multimodality biometry tool and feature extraction

XAI4Kids^®^ is a multimodality biometry tool that combines data acquisition and an AI pipeline to model heterogeneous and dynamic data/signals in relation to the target variables. Data were collected from an unmodified touch-screen tablet with an intrinsic camera (video, ~28 frames per second; digit, ~100 Hz) while participants were playing the O-Games battery. In the present paper, the two games, named *Rocket* and *Connect*, were included in the analysis. Each game was divided into challenges (11 for *Rocket* and six for *Connect*). Time-series data were analyzed for each challenge, which themselves represent a higher-order time series for each game.

Seven emotion features (**Table 1**) were predicted per frame from video processing using a single neural network that was pre-trained on face identification and fine-tuned for facial expression recognition on static images from AffectNet (38). Four eye-tracking features (**Table 1**) were extracted with a velocity-threshold identification (I-VT), a method that separates fixation and saccade points based on their point-to-point velocities (39). Using the touch data and kinetic information collected from the tablet, a set of five digit-tracking features (touch events, **Table 1**) were computed representing the participant’s motor behaviors (40).

### Statistical analysis

Databasing, statistical software, and analysis implementation were programmed in Python using the *Pandas* library (41). Statistical analysis used the *Statsmodel* and *Pingouin* packages (42). Graphs presented in this paper were plotted using the *matplotlib* (43) and *seaborn* packages (44). The Holm–Bonferroni sequential correction (45) was used to correct for multiple comparisons (46).

Extracted biometric features (emotion-, digit-, or eye-tracking) were statistically modeled as time series using a moving-average (MA) model that uses past values of the series itself and relies on a series of past errors. Indeed, since biometric features were collected as a function of the behavior of each child and their related performances at the psychometric tasks, the assumption is that the past values of the biometric values have a linear relationship with the current values. Extracted biometric features have a very high-dimensionality vector representing the data for one participant: *n* features, by 100 bins, for each *c_i_* challenge. Biometric features and bins were merged into a single dimension, accounting for a time series, consisting of a vector of *n* * 100 second-order features per participant.

Principal component analysis (PCA) was then performed on every single dimension to identify for each gamified task the three most informative components that were called “*biometric construct*” as an objective time-series measurement of biometric features that cannot be directly observed.

To reduce selection bias and improve representativeness and statistical power (overfitting), PCAs were fitted on an internal dataset of 63 children who underwent the same study protocol. The obtained eigenvectors were then applied to *biometric constructs* of the EPIDIA4Kids cohort to ensure the same dimensionality reduction.

Statistical relationships between biometric constructs and multidimensional assessment measures were examined using linear mixed models that allow for fixed and random effects to account for the effect of each O-Games challenge as well as the effects of biometric constructs on multidimensional assessment measures. O-Games challenges were set as a random effect, and each biometric construct had a fixed effect. Regression model fitting was performed for each ADHD-related symptomatology measure across challenges to account for individual variability in gaming performance. Targeted variables and explanatory variables are listed in **Table 1**. Parameter estimation was conducted using restricted maximum likelihood (REML). The model’s goodness of fit was assessed using R-squared and log-likelihood metrics. Residual diagnostics confirmed the normality, homoscedasticity, and independence of residuals. The fixed effects of the biometric constructs showed significant predictive power, with effect sizes (Cohen D between 0.4 and 1.8 for each significant PC).

To assess multimodal over unimodal biometry, model performance was examined using the mean absolute error. The multimodal biometric construct model was considered the baseline model and referred to as 1. Values for the model for each unimodal biometric construct (emotion, digit-, or eye-tracking) were calculated as percentage increases relative to the baseline model (**Table 2**).

**Table 2:** Multimodal biometry allowed capture of more multidimensional measures of ADHD-related symptomatology.

			Connect				Rocket			
			Biometric types				Biometric types			
			Multimodal	Digit-tracking	Emotion	Eye-tracking	Multimodal	Digit-tracking	Emotion	Eye-tracking
z-Score, adjusted by age and sex	**ADHD scale**	Hyperactivity and impulsivity	1	1%	0%	−2%	1	0%	0%	0%
		Inattention	1	2%	−1%	−3%	1	0%	−1%	0%
	**CBCL**	Social problems	1	3%	3%	2%	1	0%	−2%	0%
	**EHI**	Manual laterality	1	0%	1%	2%	1	0%	−1%	0%
	**GSCA**	Achievement	1	1%	−1%	−2%	1	0%	−6%	0%
	**WISC-V**	Matrix reasoning	1	0%	−3%	0%	1	0%	−3%	0%
		Digit span	1	−1%	0%	−2%	1	0%	−13%	0%
		Letter–number sequencing	1	1%	−9%	1%	1	0%	−2%	0%
		Symbol search	1	0%	−1%	3%	1	0%	−5%	0%
		Coding	1	1%	−8%	1%	1	0%	−11%	0%
		Cancellation	1	3%	1%	−1%	1	0%	−8%	0%
		Block design	1	1%	−6%	−4%	1	0%	−6%	0%
		Similarities	1	−2%	−2%	−1%	1	0%	−6%	0%
		Vocabulary	1	1%	0%	−2%	1	0%	0%	0%
		Comprehension	1	2%	−1%	−3%	1	0%	−1%	0%

The mean absolute error (MAE) was used as a critical indicator for comparing the performance of each biometric model. Comparative increased performance analysis (expressed in increased percentage) was performed between unimodal biometric model and the multimodal model, which was set as the baseline model (1).

CBCL, Child Behavior Checklist; ADHD, attention-deficit hyperactivity disorder; EHI, Edinburgh Handedness Inventory; GSCA, Grit Scale for Children and Adults; WISC-V, Wechsler Intelligence Scale for Children^®^ Fifth Edition. When p-value survived Holm-Bonferroni for mulitple comparison, values are marked in bold.

## Results

### Participant characterization

Twenty-four children (11 girls and 13 boys; mean age 10.55 years ± 1.69) were enrolled in the study as the database in December 2023. No sex difference was observed in socio-economic status or manual laterality (**Table 3**). Among neuro-assessments and standardized questionnaires, girls scored lower than boys only on the *Inattention* score of the ADHD scale (p = 0.04; F = 4.7), while boys tended to perform worse on the *Cancellation* subscale of the *WISC-V* battery than girls (p = 0.05; F = 4.26). These suggest that the children were well-matched in sex, age, and ADHD-related symptomatology.

**Table 3:** Participant characteristics.

		Girls (n = 11) Mean (std)	Boys (n = 13) Mean (std)	Exact p-value, F value, or χ^2^
	Age at assessment (years)	10.44 (1.6)	10.83 (1.82)	0.79 (0.07)
	Household income *(1 = 0–3k€, 6 = 3–37€, and 12 = ≥7k€ monthly)*	3.11 (1)	3.5 (1)	0.57 (0.35)
	Highest education (*7 = professional, 4 = high school graduate, and 1 = <7 years of school)*	5.89 (1)	6.17 (2)	0.76 (0.10)
	Highest occupation (*7 = higher executives, 4 = clerical and sales workers, and 1 = unskilled employee)*	5.0 (1)	5.66 (2)	0.45 (0.59)
	EHI, manual laterality *(raw score)*	200 (263)	223 (259)	0.84 (0.04)
z-Score, adjusted by age and sex	ADHD scale, inattention	0.48 (0.84)	0.40 (1.11)	**0.04 (4.70)**
	ADHD scale, hyperactivity and impulsivity	−0.17 (1.1)	0.18 (1.05)	0.43 (0.65)
	Social problems scale (CBCL)	−0.09 (0.99)	0.35 (0.91)	0.27 (1.29)
	GSCA, achievement	−0.60 (0.91)	−0.04 (0.84)	0.13 (2.42)
	WISC-V	(n = 11)	(n = 12)	
	Matrix reasoning	−0.17 (1.18)	0.16 (0.88)	0.46 (0.57)
	Digit span	0.16(1.16)	0.13 (1.07)	0.52 (0.42)
	Letter–Number sequencing	−0.004 (1.24)	0.003 (0.86)	0.98 (0.00)
	Symbol search	0.25 (1.21)	−0.25 (0.78)	0.29 (1.20)
	Coding	0.24 (1.16)	−0.45 (1.07)	0.17 (2.0)
	Cancellation	0.38 (0.73)	−0.65 (1.41)	**0.05 (4.26)**
	Block design	0.18 (1.16)	−0.17 (0.91)	0.45 (0.59)
	Similarities	−0.02 (0.73)	0.02 (1.27)	0.94 (0.01)
	Vocabulary	−0.28 (0.85)	0.26 (1.14)	0.24 (1.47)
	Comprehension	0.20 (0.79)	−0.17 (1.19)	0.42 (0.68)

Student’s t-tests and Mann–Whitney U tests when variables are non-parametric were performed for continuous variables. Chi-squared tests were used for categorical variables. The z-scores were adjusted for sex and age based on a proprietary dataset of 202 children aged 7 to 15 years. Results with p-values between 0.05 and 0.001 were considered a trend and significant when the p-value survived Holm–Bonferroni for multiple comparisons (marked in bold).

CBCL, Child Behavior Checklist; ADHD, attention-deficit hyperactivity disorder; EHI, Edinburgh Handedness Inventory; GSCA, Grit Scale for Children and Adults; WISC-V, Wechsler Intelligence Scale for Children^®^ Fifth Edition.

### Effects of sex and age on biometric features

Age- and sex-based differences are crucial covariates in brain function and psychopathology research (47). To account for them and minimize further bias, we examined whether these two variables alter biometric measurement. We showed that biometric features exhibited no age or sex effect in both gamified tasks. We found a sex effect only in touch area (p_(adjusted for Holm–Bonferroni correction)_ = 0.001) and touch duration (p_(adjusted for Holm–Bonferroni correction)_ = 0.001), i.e., digit-tracking features, in one of the challenges of the *Rocket* task and *Connect* task, respectively. All biometric features were then not adjusted for age and sex in contrast to neuro-assessments and standardized questionnaires.

### Multimodal biometric time-series constructs to reflect ADHD-related symptoms

Sixteen biometric features (**Table 1**) were collected from video and touchscreen events while children were playing on gamified tasks. They were extracted for the eye-, emotion-, and digit-tracking analyses to capture simultaneously behavioral, emotional, and cognitive states to obtain a multidimensional clinical picture of each child. Since trajectories, i.e., time-series data, reflect better abilities of children than just aggregated point scores, these biometric features were structured as a time-series construct per type of biometry modalities (eye-, emotion-, and digit-tracking) and called *biometric constructs*.

A PCA with all biometric constructs was performed per gamified task to extract the most informative components, named *multimodal biometric construct component* (MBC). Effects of the MBC were assessed on neuro-assessments and standardized questionnaires using both linear model and robust linear models (**Supplementary Table 1**). Both models showed that for *Connect*, MBC1 was a predictor of *Digit* sp*an* (p < 0.003) and *Letter–number sequencing* (p = 0.0001–0.04) as measures of the working memory, of *Symbol search* (p < 0.002), and to a lower extent of *Coding* (p = 0.02) and *Cancellation* (p = 0.01–0.04) as measures of processing speed. It was also found that the MBC1 has an effect on the *Inattention* score (ADHD scale *Inattention*, p = 0.04) and the *Achievement* score [Grit Scale for Children and Adults (GSCA); p = 0.04]. A strong effect on *Block design* (p = 0.003) was found only with the linear model, while MBC3 had an effect only on *Social problems* (CBCL, p = 0.007) using the robust linear model. Altogether, these results strongly suggest that the multimodal biometric constructs are objective measurements capturing ADHD symptomatology.

### Gamified-task specificities allow to capture multiple neuropsychopathological measures

Cognitive performance and rating scales, such as the WISC-V (37) alone, cannot lead to an ADHD diagnosis (4, 48), but they are commonly used in clinical practice (49). They can also provide a comprehensive assessment of ADHD-related symptomatology to align with a transdiagnostic approach. Gamified tasks were then designed to trigger several behavioral, cognitive, and emotional states in a reduced time. Using linear model regression (**Table 4**), it was found that the *Connect* task allowed predictions for working memory scores (p ≤ 0.001), for *Symbol search* (p < 0.0001), and “at lower extent” for *Inattention* (p = 0.04) and *Achievement* (p = 0.04) abilities. The *Rocket* task allowed specific predictions for processing speed as measured by *Coding* and *Cancellation* (p ≤ 0.001), *Manual laterality* [Edinburgh Handedness Inventory (EHI); p = 0.002], and *Vocabulary* (p = 0.003). Independently of the gamified tasks, each MBC1 predicted only *Block design* (p ≤ 0.003) that reflects visuo-spatial reasoning. These indicated that biometric constructs could predict several neuropsychopathological measures related to ADHD symptomatology that were triggered by the proprieties of each gamified task.

**Table 4:** Linear Model regression for significance of each multimodal biometric construct component (MBC) on neuro-assessments and standardized questionnaires for both *Rocket* and *Connect* gamified tasks.

			Connect			Rocket		
			Multimodal biometric construct component					
			MBC1	MBC2	MBC3	MBC1	MBC2	MBC3
z-Score, adjusted by age and sex	**ADHD**	Hyperactivity and impulsivity	0.4	0.35	0.94	0.392	0.863	0.61
		Inattention	0.04	0.99	0.70	0.777	0.804	0.534
	**CBCL**	Social problems	0.07	0.84	0.60	0.414	0.261	0.735
	**EHI**	Manual laterality	0.60	0.43	0.07	**0.002**	0.833	0.007
	**GSCA**	Achievement	0.04	0.09	0.59	0.054	0.095	0.159
	**WISC-V**	Matrix reasoning	0.32	0.41	0.84	0.648	0.912	0.539
		Digit span	**<0.0001**	**<0.0001**	0.25	0.091	0.685	0.742
		Letter–number sequencing	**0.001**	0.24	0.89	**<0.0001**	0.073	0.43
		Symbol search	**<0.0001**	0.41	0.15	0.967	0.909	0.366
		Coding	0.02	0.50	0.10	**<0.0001**	0.336	0.332
		Cancellation	0.01	0.71	0.50	**<0.0001**	0.938	0.054
		Block design	**0.003**	0.31	0.38	**0.001**	0.738	0.207
		Similarities	0.20	0.53	0.16	0.982	0.455	0.064
		Vocabulary	0.23	0.2	0.88	**0.003**	0.583	0.552
		Comprehension	0.34	0.16	0.07	0.486	0.53	0.067

p-Values between 0.05 and 0.001 were considered a trend and significant when the p-value survived Holm–Bonferroni for multiple comparisons (marked in bold).

CBCL, Child Behavior Checklist; ADHD, attention-deficit hyperactivity disorder; EHI, Edinburgh Handedness Inventory; GSCA, Grit Scale for Children and Adults; MBC, Multimodal Biometric Construct Component; WISC-V, Wechsler Intelligence Scale for Children^®^ Fifth Edition.

### Multimodality biometry to develop a multidimensional screening

The goal is to achieve the optimal biometric construct model to predict a multidimensional screening for ADHD-related symptomatology in children and adolescents. The mean absolute error (MAE) is a critical indicator for comparing the performance of each biometric model. **Table 2** presents a comparative analysis of performances between the multimodal and unimodal biometric models in which the multimodal model was set as the baseline model.

First, a multimodal biometric model could enhance performance to predict each of the neuropsychopathological measures but not systematically. In the *Rocket* task, unimodal models for digit- and eye-tracking had similar performances to multimodal biometric models independently of the neuropsychopathological measures, while the unimodal model for emotion (−13% to 0%) had systematically equal or better performance than multimodal biometric model for each measure. In the *Connect* task, the multimodal biometric model had better performance overall than the unimodal digit-tracking model (−1% to 3%) but worse performances over the unimodal model for emotion (−9% to 3%) and eye-tracking (−4% to 3%) dependently to the neuropsychopathological measures.

Altogether, these results suggest that multimodal biometry is required for capturing multidimensional measures of ADHD-related symptomatology in children and adolescents. However, the performance of multimodal biometric models can be lower than that obtained from unimodal biometric models.

## Discussion

These preliminary results from the EPIDIA4Kids study showed that multimodality biometry provides insights into brain function in children aged 7 to 12 years old. Biometrics may ultimately serve as a viable candidate for an objective measurement to screen for multidimensional ADHD symptomatology in children.

ADHD has a complex clinical presentation whose symptoms are highly variable including age and sex. Indeed, sex- and gender-specific differences in health exist largely due to genetic and hormonal influences of biological sex, hereby influencing physiology and disease and thus biometrics. Here, we showed that most biometric features extracted using machine learning models are not influenced by age and sex during this period of age. Further validation will be needed on a larger cohort to examine whether this statement stands. In line with previous research (24, 50), touch duration and touch area were correlated with age, suggesting that biometric constructs have a higher potential to assess neurodevelopmental symptomatology while accounting for fewer cofounders than the current assessments within this age range (neuropsychological tests or clinical questionnaires).

Furthermore, we found that biometric constructs predict performances of specific tasks that were shown mainly impaired in children and adolescents with ADHD, i.e., working memory (51, 52) and processing speed (53, 54). More specifically, several studies have reported that ADHD children have lower scores on working memory and processing speed indexes among the whole subtest battery of the WISC using its different versions (55). Additionally, we showed a specific prediction of the biometric construct for the *Digit* sp*an* subtest: performances were lower in ADHD children compared to healthy children (55). Interestingly, working memory was predicted by biometric construct mainly with the *Connect* task and processing speed with the *Rocket* task, while visuo-spatial abilities were predicted specifically in both gamified tasks. Verbal comprehension and fluid reasoning were not predicted by any of the biometric constructs. These findings suggest that biometric constructs have potential value as biomarkers for ADHD-related symptomatology where the design of gamified tasks plays a crucial role in triggering specific brain functions. However, predictions for subtest scores and indexes should be interpreted with caution since they assess symptoms at one time-point rather than clinical deficits, and they cannot reflect causality but correlations. For instance, we found that a biometric construct predicts processing speed and the *Vocabulary* subtest in the same *Rocket* task. Since verbal comprehension is highly correlated with processing speed in ADHD children (56), we cannot rule out that prediction for processing speed in *Rocket* tasks is not mediated by deficits in vocabulary performance. Altogether, biometric constructs can serve as a screening method at a large scale in children, but longitudinal studies will be needed to understand clinical causality among those biometric construct predictions.

Finally, we showed that multimodality biometry is required to capture multi-dimensional measurements of ADHD symptomatology in a limited time. Previously, eye-tracking features were reported to provide information on higher-order brain processes including memory and attention in ADHD individuals as measured by changes in gaze fixation, saccadic movements (23), or longer reaction times and errors in the direction of anti-saccadic movements (21). Digit-tracking using touchscreen events such as touch duration and the number of clicks was described as a reliable tool for quantifying attention (25) or visual motor skills (24) in ADHD children. Likewise, evidence revealed that facial emotion recognition can be used in the evaluation of children with ADHD (26–28). However, to our knowledge, none has attempted to examine whether these biometric features all collected from an unmodified tablet can serve as objective measures for a multidimensional assessment of ADHD-related symptomatology.

We found that multimodal biometric measures predicted specifically working memory and processing speed abilities that are mainly impaired in ADHD children. We should point out that unimodal biometric models outperformed sometimes the multimodal models for a specific psychopathological score. However, multimodality is required to obtain a comprehensive multidimensional picture of children. Biometric feature identification and selection will help optimize the prediction of multidimensional psychopathological measurements, but they are out of the scope of the present study.

### Limitations

The preliminary results represent thus a notable step toward objective measurements based on multimodality biometry for screening multiple dimensions of ADHD symptomatology in children. However, most participants were healthy children with no history of neurological disorders. The preliminary aim of the present study was to first test our hypothesis on the potential of multimodality biometry as a viable and scalable transdiagnostic approach for screening ADHD symptomatology. A strong tension exists between balancing the desire to minimize heterogeneity (“noise”), which can mask the effect of interest, and the desire to generate data that are generalizable to a broader population. We chose to narrow eligibility criteria to limit the variability in a study population and control for confounding factors. Narrowing eligibility criteria will most probably diminish the understanding and applicability of the findings in real-world ADHD populations, but this step is crucial to move forward. Increasing the number of participants diagnosed with ADHD is required to establish generalizability with rigorous clinical validation.

As a secondary judgment criterion, multimodality approaches bring high potential for improving access to trained specialists as well as diagnosis support at cost-efficiency (57–59). Recent machine learning techniques could handle heterogeneous data from multimodal sources for prediction (60–62), but combining data from multiple sources can hurt model performance (63) and increase the risk of incurring biases (64). Given this, we applied PCA technology, which appears the appropriate approach to minimize data dimensionality, thereby stabilizing subsequent modeling (65) to mitigate biases.

Another limitation is to account for the inherent complexity of health data that encompass many domains (social, biological, environmental, and genetic) that influence health. Multimodal machine learning can leverage different types of data and find patterns in and between modalities to improve prediction performance (66) using transformer architectures. However, training of supervised machine learning model relied on existing samples, and no database with images of children with ADHD is available. A promising aspect of transformers is the ability to learn meaningful representation with unlabeled data, which is paramount in biomedical artificial intelligence given the limited and expensive resources needed to obtain high-quality labels. Diagnostic testing tools such as those based on digital technology, i.e., multimodal biometry, would then provide for global mental and medical states of individuals in a shorter time but will not replace the professionals’ experience and observation time (29, 67). Such scales or tools are complementary to them, as they will be helpful in diagnosing and predicting the full spectrum of ADHD symptoms by accelerating and improving medical information and history retrievals. Knowledge of clinical expertise and technologies is required to gather clinical evidence for such digital tools. Diagnosis of ADHD will stay primarily a clinical one where clinicians/professionals are at the core of the whole process. However, there is a shortage of medical professionals in which 40% of medical doctors are retiring or will retire by 2030. Therefore, remote and digital assessments would be valuable tools in routine care, but it is essential to account for patients and relatives who are digitally disadvantaged and cannot access remote technologies (68). Low- and middle-income families or households have often lower digital literacy and are less likely to access digital psychiatric services (69). To minimize such social inequities, the XAI4Kids^®^ system was developed for ADHD assessments at a large scale in a natural setting without the need for additional cost-limiting equipment.

## Conclusions

Altogether, evidence strongly suggests that the XAI4Kids^®^ system combining biometric measures and transdiagnostic approaches may provide compelling alternatives in capturing the multiple dimensions of ADHD symptomatology in the pediatric population and at a large scale. Multimodality data and modeling have the goal of addressing urgent issues such as easing access to child specialists due to limited resources.

Future work focuses on conducting a comprehensive and rigorous validation with clinically diagnosed ADHD participants to ensure reliability in clinical settings but also apprehend differences in ADHD presentation and comorbidities. Lastly, the integration of XAI4Kids^®^ and its efficiency along the existing clinical workflow will be examined to tailor screening and diagnosis in primary care.

## Data Availability

The datasets presented in this article are not readily available because it was not included in the ethics approval to submit the datasets.
