# Supplementary material for: Preliminary results of the EPIDIA4Kids study on brain function in children: multidimensional ADHD-related symptomatology screening using multimodality biometry
Source: Front Psychiatry. 2025 Mar 17;16:1466107. doi: 10.3389/fpsyt.2025.1466107 (PMC11955964; doi:10.3389/fpsyt.2025.1466107)
Supplement: Supplementary file 1 [file Table1.docx]

**e-Table 1. Optimum Models for Significance of each Multimodal Biometry Construct Component (MBC) on neuro-assessments and standardized questionnaires for the *Connect* gamified task.**

Results with p-values between 0.05 and 0.001 were considered as a trend, and as significant when the p-value survived Holm-Bonferroni for multiple comparison (marked in bold).

List of Abbreviations. CBCL: Child Behavior Checklist; ADHD: Attention Deficit Hyperactivity Disorder; EHI: Edinburgh Handedness Inventory; GSCA: Grit Scale for Children and Adults; MBC: Multimodal Biometric Construct Component; WISC-V: Wechsler Intelligence Scale for Children® Fifth Edition

|  | | | **Statistical model** | | | | | |
| --- | --- | --- | --- | --- | --- | --- | --- | --- |
|  | | | **LINEAR MODEL** | | | **ROBUST LINEAR MODEL** | | |
|  | | | Multimodal Biometric Construct Component | | | | | |
|  | | | MBC1 | MBC2 | MBC3 | MBC1 | MBC2 | MBC3 |
| z-score, adjusted by age and sex | **ADHD** | Hyperactivity & Impulsivity | 0,4 | 0,35 | 0,94 | 0,35 | 0,44 | 0,80 |
|  |  | Inattention | 0,04 | 0,99 | 0,70 | 0,05 | 0,37 | 0,12 |
|  | **CBCL** | Social Problems | 0,07 | 0,84 | 0,60 | 0,66 | 0,06 | 0,007 |
|  | **EHD** | Manual Laterality | 0,60 | 0,43 | 0,07 | 0,58 | 0,37 | 0,35 |
|  | **GSCA** | Achievement | 0,04 | 0,09 | 0,59 | 0,04 | 0,74 | 0,28 |
|  | **WISC-V** | Matrix reasoning | 0,32 | 0,41 | 0,84 | 0,96 | 0,38 | 0,22 |
|  |  | Digit span | **<0.0001** | **<0.0001** | 0,25 | **0,003** | 0,06 | 0,33 |
|  |  | Letter-Number Sequencing | **0,001** | 0,24 | 0,89 | 0,04 | 0,31 | 0,89 |
|  |  | Symbol search | **<0.0001** | 0,41 | 0,15 | **0,002** | 0,06 | 0,45 |
|  |  | Coding | 0,02 | 0,50 | 0,10 | 0,03 | 0,02 | 0,67 |
|  |  | Cancellation | 0,01 | 0,71 | 0,50 | 0,04 | 0,01 | 0,94 |
|  |  | Block Design | **0,003** | 0,31 | 0,38 | 0,17 | 0,47 | 0,99 |
|  |  | Similarities | 0,20 | 0,53 | 0,16 | 0,32 | 0,17 | 0,66 |
|  |  | Vocabulary | 0,23 | 0,2 | 0,88 | 0,34 | 0,08 | 0,16 |
|  |  | Comprehension | 0,34 | 0,16 | 0,07 | 0,75 | 0,80 | 0,96 |
